# Identification of differentially expressed genes in fibroblasts derived from patients with Dupuytren's Contracture

**DOI:** 10.1186/1755-8794-1-10

**Published:** 2008-04-23

**Authors:** Latha Satish, William A LaFramboise, David B O'Gorman, Sandra Johnson, Benjamin Janto, Bing Siang Gan, Mark E Baratz, Fen Z Hu, J Christopher Post, Garth D Ehrlich, Sandeep Kathju

**Affiliations:** 1 Center for Genomic Sciences, Allegheny-Singer Research Institute, Allegheny General Hospital, Pittsburgh, PA, USA; 2 Department of Pathology, University of Pittsburgh, PA, USA; 3 Cell and Molecular Biology Laboratory of the Hand and Upper Limb Centre, London, Ontario, Canada; 4 Division of Upper Extremity Surgery, Department of Orthopaedics, Allegheny General Hospital, Pittsburgh, PA, USA; 5 Department of Microbiology and Immunology, Drexel University College of Medicine, Allegheny Campus, Pittsburgh, PA, USA; 6 Department of Otolaryngology, Drexel University College of Medicine, Allegheny Campus, Pittsburgh, PA, USA; 7 Department of Human Genetics, Drexel University College of Medicine, Allegheny Campus, Pittsburgh, PA, USA

## Abstract

Dupuytren's contracture (DC) is the most common inherited connective tissue disease of humans and is hypothesized to be associated with aberrant wound healing of the palmar fascia. Fibroblasts and myofibroblasts are believed to play an important role in the genesis of DC and the fibroproliferation and contraction that are hallmarks of this disease. This study compares the gene expression profiles of fibroblasts isolated from DC patients and controls in an attempt to identify key genes whose regulation might be significantly altered in fibroblasts found within the palmar fascia of Dupuytren's patients. Total RNA isolated from diseased palmar fascia (DC) and normal palmar fascia (obtained during carpal tunnel release; 6 samples per group) was subjected to quantitative analyses using two different microarray platforms (GE Code Link™ and Illumina™) to identify and validate differentially expressed genes. The data obtained was analyzed using The Significance Analysis of Microarrays (SAM) software through which we identified 69 and 40 differentially regulated gene transcripts using the CodeLink™ and Illumina™ platforms, respectively. The CodeLink™ platform identified 18 upregulated and 51 downregulated genes. Using the Illumina™ platform, 40 genes were identified as downregulated, eleven of which were identified by both platforms. Quantitative RT-PCR confirmed the downregulation of three high-interest candidate genes which are all components of the extracellular matrix: proteoglycan 4 (PRG4), fibulin-1 (FBLN-1) transcript variant D, and type XV collagen alpha 1 chain. Overall, our study has identified a variety of candidate genes that may be involved in the pathophysiology of Dupuytren's contracture and may ultimately serve as attractive molecular targets for alternative therapies.

## Background

Dupuytren's contracture (DC) is the most common inherited disease of connective tissue in humans [1] and an autosomal dominant form of the disease was recently mapped to the long arm of chromosome 16 [2]. The disease is characterized by the appearance of small nodules of hyperproliferative cells within the palmar fascia that, over time, give rise to large bands of contracted, collagen-rich fibrotic tissue (diseased cords), a hallmark of the disease [3,4]. If left untreated, this disease may impose severe limitations on hand function. It is a familial disorder that is highly prevalent in individuals of Northern European extraction [5,6] and is observed less frequently among other ethnicities [7]. The manifestations of Dupuytren's are usually noticeable between the ages of 40 to 60 and with a higher incidence in men than in women [8].

Although the pathogenesis of DC disease has not been fully explained it is clear that genetics plays an important role; traumatic factors may also be important and may explain the male gender bias of the disease. In addition, a number of metabolic conditions that negatively affect wound healing processes in general have been statistically associated with DC including: diabetes mellitus (8%), alcoholism (10%), smoking, and HIV infection [9-12]. Finally, there is a puzzling connection with epilepsy (2%).

The mainstay of treatment is surgery, but no specific surgical approach has proved to be consistently more effective than others at curing this condition as the trauma associated with surgery itself can lead to recurrence. Possible alternatives to surgery include injection of steroids, γ-interferon [13,14], use of creams based on vitamin E, dimethylsulphoxides, drugs inducing hypo-uricaemia, ultrasonic therapy [15] and clostridial collagenase injection [16]. However, these medical treatments appear to be either temporary alternatives to surgical intervention with only limited success at best, or are still under clinical assessment.

Previous studies have identified dysregulation among multiple structural proteins in DC, including: type I and type III collagens; the extracellular matrix (ECM) proteins fibronectin, tenascin C, and laminin; as well as matrix metalloproteinases in the diseased fascial cords [17,18]. Previous studies have also shown that a number of signaling molecules such as transforming growth factor β (TGFβ), epidermal growth factor (EGF) and platelet derived growth factor (PDGF) are differentially regulated in DC [19,20]. No specific causative gene has yet been identified and identification of susceptibility loci may help to unravel the pathogenesis of this common disease. Our laboratory has investigated the pattern of inheritance of DC in a large Swedish family and has shown that DC is inherited as an autosomal dominant disorder with incomplete penetrance by the end of the fifth decade [2]. A genome-wide scan at a resolution of ~8 cM for all autosomes established linkage to a single 6 cM region between markers D16S419 and D16S3032 on chromosome 16q. In contrast, Bayat et al., (2005) [21] identified a heteroplasmic mitochondrial mutation associated with DC. These investigators have also identified associations between polymorphisms in the human TGFβRI and Zf9 genes with DC [22,23] raising the possibility that DC may be the common result of multiple disparate genetic lesions.

Previously investigators have conducted differential gene expression profiling of tissues collected from Dupuytren's patients (using normal palmar fascia as controls [24-26]). In the present study we compare the gene expression patterns in fibroblasts derived from DC and carpal tunnel patients, as fibroblasts are believed to play an important role in the genesis of DC and fibroproliferation is one of the hallmarks of the disorder [27,28]. Since DC has been hypothesized to result from an impaired wound healing response, cellular elements such as myofibroblasts (primarily derived from fibroblasts), which are implicated both in the contraction of wound granulation tissue and the scar-like disease cords that define DC, are attractive targets for study. Fibroblasts/myofibroblasts actively control both extracellular matrix remodeling and scar deposition; processes which are central to wound healing and may be dysregulated in DC. In this context, the present study was designed as an attempt to identify key genes whose expression is significantly altered in the principal cell type of interest during the development of Dupuytren's contracture. We have followed the recommendations of the Microarray Quality Control Consortium (MAQC) [29], which recommends the use of multiple microarray platforms for the quantitative characterization of gene expression. Accordingly, we used both the GE Code Link™ and Illumina™ systems for cross validation.

## Methods

### Cell Culture

Dupuytren's contracture cords were surgically resected at St Joseph's Hospital and normal palmar fascia was obtained from patients undergoing carpal tunnel release within the Hand and Upper Limb Centre (HULC) clinic, London, Ontario, Canada. Primary fibroblasts were isolated from freshly surgically resected DC cord or carpal tunnel resection-derived normal palmar fascia explants in starter medium containing α-MEM-medium (Invitrogen Corporation, Carlsbad, CA) supplemented with 10% fetal bovine serum (Gemini Bioproducts, West Sacramento, CA) and 1% antibiotic-antimycotic solution (Sigma-Aldrich, St Louis, MO) in the Cell and Molecular Biology Laboratory of the HULC. Fibroblasts were identified by their characteristic spindle shaped morphology. Cells were grown in 10 cm^2 ^plates and, upon reaching 80% confluence, were lysed using RLT-lysis buffer to extract total RNA (Qiagen, Valencia, CA). RNA quality was assessed by OD_A260/280 _using an ND-1000 spectrophotometer (Nanodrop Technologies Inc, Wilmington, DE) and by capillary electrophoresis with the Agilent 2100 bioanalyzer (Agilent Technolgies, Inc. Santa Clara, CA). All subjects provided written informed consent under the approval of the Health Sciences Research Involving Human Subjects (HSREB) and specimens were collected in strict compliance by the physician performing the surgeries.

### Micorarray Expression Analyses using Illumina™ and GE CodeLink™ Bioarray Systems

Microarray experiments were performed to identify genes that are differentially expressed in fibroblasts derived from DC-affected palmar fascia versus those derived from normal fascia. 500 ng samples of total RNA, isolated from palmar fascia fibroblasts, derived either from carpal tunnel release (CTR) controls (N = 6) or DC-affected patients (N = 6), were subjected to analyses with the CodeLink™ Human Whole Genome Bioarray System (containing ~57,000 transcript probes which includes ~45,000 well characterized transcript targets, GE Healthcare Biosciences Corp; Piscataway, NJ) and the Sentrix Human-6 Expression Beadchip (which contains more than 48,000 transcript probes, Illumina; SanDiego, CA). Total RNA was amplified, labeled and hybridized according to the manufacturers' protocols. Biotin labeled cRNA probe was prepared by a linear amplification method. After first and second strand synthesis, the cDNA served as the template for an *in vitro *transcription (IVT) reaction to produce the target cRNA. The biotin labeled cRNA (10 μg for CodeLink™ and 1.5 μg for Illumina™) was hybridized overnight in a temperature controlled shaking incubator at 37°C. Post-hybridization processing included a stringent wash to remove unbound and non-specifically hybridized target molecules, a staining step with a Cy™ 5-streptavidin conjugate (CodeLink™) or with Cy™3-streptavidin conjugate (Illumina™) followed by several non-stringent washing steps to remove unbound conjugate. Following a final rinse, the bioarrays were dried by centrifugation and scanned. The CodeLink bioarrays were read in a Lumonix-Packard scanner and the Illumina arrays were read in an Illumina Bead Array Reader. The data from both platforms were analyzed using the Significance Analysis of Microarrays (SAM) software package [30].

### Analysis of Gene Expression Data

SAM software tool v3.0 was applied to identify those genes that had statistically significant differences in expression between Dupuytren's- and carpal tunnel-derived fibroblasts [30]. Briefly, SAM computes a score for each gene that measures the strength of transcript correlation with differential expression. A threshold value of 1.03 and 1.17 was chosen for CodeLink™ and Illimina™ data respectively, as estimated by repeatedly permuting the survival times and counting the number of genes that were significant at each threshold. Missing data were handled using the K-nearest neighbors imputer (k = 2) of the SAM imputation gene.

### Network Generation and Functional Analysis Using Ingenuity Pathways Analysis

Network and functional analyses were generated through the use of Ingenuity Pathways Analysis (Ingenuity Systems). A data set containing gene identifiers and corresponding expression values was uploaded into the application. Each gene identifier was mapped to its corresponding gene object in the Ingenuity Pathways Knowledge Base. These genes, called Focus Genes, were overlaid onto a global molecular network developed from information contained in the Ingenuity Pathways Knowledge Base. Networks of these Focus Genes were then algorithmically generated based on their connectivity. The Functional Analysis of a network identified the biological functions and/or diseases that were most significant to the genes in the network. Fischer's exact test was used to calculate a *p *value determining the probability that each biological function and/or disease assigned to that network is due to chance alone. A *p *value ≤ 0.05 was considered significant. Canonical pathways analysis of the Ingenuity Pathways Analysis Library identified those pathways that were most significant to the data set.

### Real-time RT-PCR

Real-time RT-PCR-based assays were performed for three gene products of interest whose annotation suggested a plausible role in the pathogenesis of DC. These included: proteoglycan 4 (PRG4), fibulin 1 – transcript variant D, and type XV collagen. For these assays, total RNA was isolated from normal and diseased palmar fascia fibroblasts using the RNeasy Micro Kit (Qiagen Inc. USA, Valencia, CA). Reverse transcription was performed using 10 ng of total RNA with M-MLV-reverse transcriptase (In Vitrogen Corporation, Carlsbad, CA) with random primers at a concentration of 100 ng/μl. GAPDH was simultaneously assayed by quantitative RT-PCR as an endogenous invariant control for normalization. PCR amplification and detection of template was carried out using Applied Biosystems transcript-specific assays including: proteoglycan 4 – Hs00195140_m1, type XV collagen – Hs01559630_m1, fibulin 1 (transcript variant D) – Hs00197774_m1. These assay kits utilize FAM™Taqman^® ^MGB probes and a Taqman^® ^Universal PCR Master Mix, all obtained from Applied Biosystems (Foster City, CA). Each reaction mix contained template cDNA at a concentration of 15 ng/μl, and 20× final concentration of Gene Expression Mix which contains both forward and reverse primers along with the gene specific probes adjusted to a final volume of 15.0 μl. Following reaction setup, aliquots were transferred into a microAMP Optical 96-well reaction plate (Applied Biosystems). Samples were vortexed, briefly centrifuged, and sealed with an optical adhesive cover (Applied Biosystems). The universal thermal cycling protocol suggested by the Applied Biosystems protocol was performed using the ABI Prism ^® ^7900 HT sequence detection system using the following cycling conditions: enzyme activation at 95°C for 10 minutes, followed by 40 cycles of two-temperature PCR at 95°C for 15 seconds and 60°C for 1 minute. Using the comparative critical cycle (C_t_) method the expression levels of the target genes were normalized to the GAPDH endogenous control and the relative abundance was calculated. Data were analyzed using the 7900 HT SDS software version 2.1 provided by Applied Biosystems.

### Statistical Analysis

The real time RT-PCR data were analyzed using an unpaired two-tailed Student's *t *test to determine the statistical significant difference in gene expression levels between control and diseased fibroblasts. A *p *value ≤ 0.05 was considered significant.

## Results

The present study was designed to identify differences in the gene expression patterns between primary Dupuytren's contracture-derived fibroblasts and primary carpal tunnel-derived control fibroblasts. This was achieved employing two different microarray platforms, the GE CodeLink™ and Illumina™, using RNA extracted from primary cultures of carpal tunnel- and DC-derived fibroblasts within passages 2–5 of tissue culture (P2–P5). Furthermore, several high interest candidate genes identified by analysis of the microarray data were further validated using quantitative real time RT-PCR, and the online Ingenuity Knowledge Based Analysis system was used to identify signaling pathways that could serve as potential therapeutic targets to limit the recurrence of DC disease.

### Microarray

SAM software (v3.0) [30] was applied to global median normalized data from CodeLink™. The data from the Illumina™ analysis was median normalized and background values were subsequently removed in accordance with the manufacturer's protocol before analysis using SAM. Genes that were differentially expressed between the control and diseased fibroblasts in at least four of six samples were included in the analysis. The % coefficient of variation (CVs) of the GE CodeLink ™ and Illumina ™ data were calculated and the results were 16.9% and 17.74% for the controls (carpal tunnel derived fibroblasts) and 17.1% and 12.13% for the test samples (Dupuytren's contracture cord derived fibroblasts) that were then analyzed using SAM. This incorporated all 47,290 (GE CodeLink™) and 48,000 (Illumina™) transcript probes in the genome wide arrays. Thus the two groups were comparable. A two-class unpaired comparison of gene expression data with SAM was performed at a false discovery rate (FDR %) of 8.1% (Delta value of 1.03) with CodeLink™ data; for Illumina™ SAM was performed at a FDR % of 1.0% (Delta value of 1.17).

We identified 69 genes and ESTs that were differentially expressed using the CodeLink™ arrays and 40 using the Illumina™ arrays. Of the 69 CodeLink-identified genes, 18 were upregulated and 51 were downregulated (including ESTs and cDNA clones of unknown function). All 40 genes and ESTs identified using the Illumina™ platform were downregulated (Table 1, 2 &3). A comparison of the data from the two platforms identified 11 genes that were identified as being downregulated by both systems.

**Table 1:** Genes upregulated in palmar fascia fibroblasts obtained from Dupuytren's Contracture using CodeLink™ platform.

	**Gene ID**	**Gene Name**	**Fold Change**
1.	NM_014893	Neuroligin 4, Y-linked (NLGN4Y)	10.4
2.	NM_004653	Jumonji, AT rich interactive domain 1D (RB2-like) (JARID1D)	9.4
3.	NM_003411	Zinc finger protein, Y-linked (ZFY)	8.9
4.	NM_138963	Ribosomal protein S4, Y-linked 2 (RPS4Y2)	6.7
5.	NM_004660	DEAD (Asp-Glu-Ala-Asp) box polypeptide 3, Y-linked (DDX3Y)	6.3
6.	NM_001008	Ribosomal protein S4, Y-linked 1 (RPS4Y1)	6.1
7.	NM_002527	Neurotrophin 3 (NTF3)	2.9
8.	NM_006558	KH domain containing, RNA binding, signal transduction associated 3 (KHDRBS3)	1.8
9.	NM_033546	Myosin regulatory light chain MRLC2 (MRLC2)	1.3
10.	NM_198833	Serine (or cysteine) proteinase inhibitor, clade B (ovalbumin), member 8 (SERPINB8)	1.2

***cDNA of Unknown Biological Function***			

1.	AA156287	zo33b08s1 Stratagene colon (#937204) cDNA clone IMAGE:588663 3'	74.62
2.	BQ924832	AGENCOURT_8840265 Lupski_sciatic_nerve cDNA clone IMAGE:6205036 5'	9.46
3.	BX648643	mRNA; cDNA DKFZp686O17106 (from clone DKFZp686O17106)	8.13
4.	Z48511	HSXGPEP11 H. sapiens XG mRNA (clone PEP11)	7.21
5.	AI674196	wc09d11x1 NCI_CGAP_Pr28 cDNA clone IMAGE:2314677 3'	6.85
6.	H70730	yu69e10r1 Weizmann Olfactory Epithelium cDNA clone IMAGE:239082 5'	5.76
7.	AA49137	zx03d12r1 Soares_total_fetus_Nb2HF8_9w cDNA clone IMAGE:785399 5'	2.94
8.	NM_032576	Chromosome Y open reading frame 15B (CYorf15B), mRNA	2.71

SAM analysis was performed to identify the statistically significant differentially expressed genes.

**Table 2:** Genes downregulated in palmar fascia fibroblasts obtained from Dupuytren's Contracture using CodeLink™ Platform.

	**Gene ID**	**Gene Name**	**Fold Change**
1.	NM_002847	Protein tyrosine phosphatase, receptor type, N polypeptide 2 (PTPRN2), transcript variant 1	0.16
2.	NM_021602	CD79B antigen (immunoglobulin-associated beta) (CD79B), transcript variant 2	0.18
3.	NM_001831	Clusterin (complement lysis inhibitor, SP-40,40, sulfated glycoprotein 2, testosterone-repressed prostate message 2, apolipoprotein J) (CLU), transcript variant 1	0.21
***4.***	NM_004000	***chitinase-3-like 2 (CHI3L2)***	***0.22***
5.	NM_152536	FYVE, RhoGEF and PH domain containing 5 (FGD5), mRNA	0.25
6.	NM_000668	Alcohol dehydrogenase 1B (class I), beta polypeptide (ADH1B)	0.29
***7.***	NM_001831	***clusterin (complement lysis inhibitor, SP-40,40, sulfated glycoprotein 2, testosterone-repressed prostate message 2, apolipoprotein J) (CLU), transcript variant 1***	***0.30***
***8.***	NM_005410	***selenoprotein P, plasma, 1 (SEPP1)***	***0.34***
***9.***	NM_005525	***hydroxysteroid (11-beta)dehydrogenase 1 (HSD11B1), transcript variant 1***	***0.35***
10.	NM_145244	DNA-damage inducible transcript 4-like (DDIT4L)	0.37
***11.***	NM_012168	***F-box protein 2 (FBXO2), mRNA***	***0.38***
***12.***	NM_021146	***angiopoietin-like factor (CDT6)***	***0.39***
13.	U83115	Non-lens beta gamma-crystallin like protein (AIM1)mRNA	0.42
***14.***	NM_001801	***cysteine dioxygenase type1 (CDO1)***	***0.45***
**15.**	NM_005627	***serum/glucocorticoid regulated kinase (SGK)***	***0.49***
16.	NM_002928	Regulator of G-protein signaling 16 (RGS16)	0.49
***17.***	NM_000690	***aldehyde dehydrogenase 2 family (mitochondrial) (ALDH2), nuclear gene encoding mitochondrial protein***	***0.54***
18.	NM_005391	pyruvate dehydrogenase kinase, isoenzyme 3 (PDK3)	0.55
***19.***	NM_000636	***superoxide dismutase 2, mitochondrial (SOD2)***	***0.56***
20.	NM_006391	Importin 7 (IPO7)	0.57
21.	NM_030968	C1q and tumor necrosis factor related protein 1 (C1QTNF1), transcript variant 1	0.58
***22.***	NM_001353	***aldo-keto reductase family 1, member C1 (dihyrdodiol dehydrogenase 1;20-alpha (3-alpha)-hydroxysteroid dehydrogenase) (AKR1C1)***	***0.59***

***cDNA of Unknown Biological Function***			

1.	D52654	HUM084D02B Clontech human fetal brain polyA+ mRNA (#6535) cDNA clone GEN-084D02 5'	0.00
2.	AI597810	tu91f07x1 NCI_CGAP_Gas4 cDNA clone IMAGE:2258437 3' similar to contains L1.t1 L1 L1 repetitive element;	0.01
3.	H90897	yu89c07s1 Soares fetal liver spleen 1NFLS cDNA clone IMAGE:240972 3'	0.10
4.	NM_021602	CD79B antigen (immunoglobulin-associated beta) (CD79B), transcript variant 2	0.18
5.	BX094389	NCI_CGAP_Ut4 cDNA clone IMAGp998O234898	0.23
6.	NM_152536	FYVE, RhoGEF and PH domain containing 5 (FGD5), mRNA	0.25
7.	AK054990	cDNA FLJ30428 fis, clone BRACE2008941	0.31
8.	AA866107	oh53b03.s1 NCI_CGAP_GC4 cDNA clone IMAGE:1470317 3'	0.33
9.	BU624020	UI-H-FG1-bgh-h-24-0-UIs1 NCI_CGAP_FG1 cDNA clone UI-H-FG1-bgh-h-24-0-UI 3'	0.34
10.	AI186220	qd34a02x1 Soares_fetal_heart_NbHH19W cDNA clone IMAGE:1731338 3'	0.35
11.	BG117761	602350442F1 NIH_MGC_90 cDNA clone IMAGE:4445147 5'	0.40
12.	BM929454	UI-E-EJ1-aje-b-23-0-UIr1 UI-E-EJ1 cDNA clone UI-E-EJ1-aje-b-23-0-UI 5'	0.42
13.	AL117425	mRNA; cDNA DKFZp566L203 (from clone DKFZp566L203)	0.43
14.	BQ430788	AGENCOURT_7776027 NIH_MGC_68 cDNA clone IMAGE:6024295 5'	0.44
15.	AA594928	no40a05s1 NCI_CGAP_Pr23 cDNA clone IMAGE:1103120 3'	0.46
16.	H05195	yl85f06r1 Soares infant brain 1NIB cDNA clone IMAGE:45005 5'	0.47
17.	AK124841	cDNA FLJ42851 fis, clone BRHIP2005719	0.47
18.	H05195	yl85f06r1 Soares infant brain 1NIB cDNA clone IMAGE:45005 5'	0.48
19.	AL119769	DKFZp761E1224_r1 761 (synonym: hamy2) cDNA clone DKFZp761E1224 5'	0.54
20.	AI094001	qa28a03s1 Soares_NhHMPu_S1 cDNA clone IMAGE:1688044 3'	0.54
21.	AL133055	mRNA; cDNA DKFZp434J1015 (from clone DKFZp434J1015)	0.56
22.	BX094154	Soares fetal liver spleen 1NFLS cDNA clone IMAGp998P17654	0.58
23.	W67378	zd40b03r1 Soares_fetal_heart_NbHH19W cDNA clone IMAGE:343085 5' similar to SW:DHAM_HUMAN P05091 ALDEHYDE DEHYDROGENASE, MITOCHONDRIAL PRECURSOR;	0.58
24.	BM829211	K-EST0102153 S9SNU601 cDNA clone S9SNU601-57-C10 5'	0.58
25.	BM711907	UI-E-CL1-afc-i-20-0-UIr1 UI-E-CL1 cDNA clone UI-E-CL1-afc-i-20-0-UI 5'	0.59
26.	BX088944	Soares_testis_NHT cDNA clone MAGp998J113516; IMAGE:1392490	0.72
27.	BC033399	clone IMAGE:4829538	0.77
28.	CB960582	AGENCOURT_13892014 NIH_MGC_147 cDNA clone IMAGE:30343321 5'	0.79
29.	BC034566	clone IMAGE:4821877, mRNA	0.79

SAM analysis was performed to identify the statistically significant differentially expressed genes. Genes that are common to both platforms are highlighted as *Bold and Italics*.

**Table 3:** Genes downregulated in palmar fascia fibroblasts obtained from Dupuytren's Contracture using Illumina™ Platform.

	**Gene ID**	**Gene Name**	**Fold Change**
1.	NM_005807	Homo sapiens proteoglycan 4 (PRG4), mRNA.	0.08
2.	NM_020299	Homo sapiens aldo-keto reductase family 1, member B10 aldose reductase) (AKR1B10), mRNA.	0.15
3.	NM_145176	Homo sapiens solute carrier family 2 (facilitated glucose transporter), member 12 (SLC2A12), mRNA.	0.18
4.	NM_001831	***clusterin (complement lysis inhibitor, SP-40,40, sulfated glycoprotein 2, testosterone-repressed prostate message 2, apolipoprotein J) (CLU), transcript variant 1***	***0.21***
**5.**	NM_012168	***F-box protein 2 (FBXO2), mRNA***	***0.21***
6.	NM_139125	Homo sapiens mannan-binding lectin serine peptidase 1 (C4/C2 activating component of Ra-reactive factor) (MASP1), transcript variant 2, mRNA.	0.22
7.	NM_000691	Homo sapiens aldehyde dehydrogenase 3 family, memberA1 (ALDH3A1), mRNA	0.22
8.	NM_178127	Homo sapiens angiopoietin-like 5 (ANGPTL5), mRNA	0.23
9.	NM_004000	***chitinase-3-like 2 (CHI3L2)***	***0.22***
**10.**	NM_005410	***Homo sapiens selenoprotein P, plasma, 1 (SEPP1), mRNA***	***0.23***
11.	NM_001996	Homo sapiens fibulin 1 (FBLN1), transcript variant C, mRNA	0.24
12.	NM_015714	Homo sapiens G0/G1switch 2 (G0S2), mRNA	0.24
13.	NM_021146	***angiopoietin-like factor (CDT6)***	***0.24***
14.	NM_006486	Homo sapiens fibulin 1 (FBLN1), transcript variant D, mRNA	0.25
15.	NM_000956	Homo sapiens prostaglandin E receptor 2 (subtype EP2), 53kDa (PTGER2), mRNA	0.26
16.	NM_005525	***Homo sapiens hydroxysteroid (11-beta) dehydrogenase 1 (HSD11B1), transcript variant 1, mRNA***	***0.28***
17.	NM_173640	Homo sapiens R-spondin homolog (Xenopus laevis) (RSPO1), transcript variant 2, mRNA.	0.30
***18.***	NM_001801	***cysteine dioxygenase type1 (CDO1)***	***0.30***
19.	XM_352750	Homo sapiens collagen, type XIV, alpha 1 (undulin) (COL14A1), mRNA.	0.31
20.	NM_002150	Homo sapiens 4-hydroxyphenylpyruvate dioxygenase (HPD), mRNA	0.33
21.	NM_001855	Homo sapiens collagen, type XV, alpha 1 (COL15A1), mRNA.	0.33
22.	XM_166300	Homo sapiens absent in melanoma 1 (AIM1), mRNA	0.36
23.	NM_005627	***Homo sapiens serum/glucocorticoid regulated kinase (SGK), mRNA***	***0.36***
24.	NM_016564	Homo sapiens cell cycle exit and neuronal differentiation 1 (CEND1), mRNA.	0.38
25.	NM_133371	Homo sapiens myozenin 3 (MYOZ3), mRNA	0.40
26.	NM_001290	Homo sapiens LIM domain binding 2 (LDB2), mRNA.	0.41
27.	NM_003383	Homo sapiens very low density lipoprotein receptor (VLDLR), transcript variant 1, mRNA.	0.43
28.	NM_005631	Homo sapiens smoothened homolog (Drosophila) (SMO), mRNA	0.43
29.	NM_014746	Homo sapiens ring finger protein 144 (RNF144), mRNA	0.44
30.	NM_000636	***superoxide dismutase 2, mitochondrial (SOD2)***	***0.45***
**31.**	NM_000690	***aldehyde dehydrogenase 2 family (mitochondrial) (ALDH2), nuclear gene encoding mitochondrial protein, mRNA.***	***0.48***
32.	NM_002222	Homo sapiens inositol 1,4,5-triphosphate receptor, type 1 (ITPR1), mRNA	0.48
33.	NM_012329	Homo sapiens monocyte to macrophage differentiation-associated (MMD), mRNA.	0.50
34.	NM_000861	Homo sapiens histamine receptor H1 (HRH1), mRNA	0.50
***35.***	NM_001353	***aldo-keto reductase family 1, member C1 (dihydrodiol dehydrogenase 1;20-alpha (3-alpha)-hydroxysteroid dehydrogenase) (AKR1C1)***	***0.51***

***cDNA of Unknown Biological Function***			

1.	NM_024563.2	Homo sapiens chromosome 5 open reading frame 23 (c5orf23)mRNA	0.48
2.	XM_376189.1	Homo sapiens DKFZP586K1520 protein (DKFZP586K1520), mRNA	0.41
3.	NM_014859.1	Homo sapiens KIAA0672 gene product (KIAA0672), mRNA.	0.28
4.	XM_352945.1	Homo sapiens hypothetical protein FLJ37034 (FLJ37034), mRNA	0.46
5.	NM_138409.1	Homo sapiens chromosome 6 open reading frame 117 (C6orf117), mRNA	0.21

SAM analysis was performed to identify the statistically significant differentially expressed genes. Genes that are common to both platforms are highlighted as *Bold and Italics*.

We also identified several other differentially expressed genes of interest using the Illumina™ platform but not CodeLink™ platform through SAM analysis. Interestingly, direct examination of the CodeLink data set revealed that these genes were also observed to be downregulated by the CodeLink software (Table 4), albeit not sufficiently to satisfy the extremely stringent parameters of SAM analysis. Subsequent direct quantification of three genes by quantitative RT-PCR confirmed their relative underexpression in DC-derived fibroblasts, indicating that these data were consistent.

**Table 4:** mRNA levels of gene products that are significantly different in CodeLink™ gene array platform similar to Illumina™ platform.

	**Gene**	**Control**	**Diseased**	**Fold Change**	***P *value**
1.	NM_005807 Homo sapiens PRG4	791.69	60.72	0.15	0.01
2.	NM_006486 Fibulin 1(FBLN1), transcript variant D	101.28	30.97	0.30	0.007
3.	NM_001855 Collagen, Type XV, alpha 1 (COL15A1)	52.92	19.59	0.37	0.01
4.	NM_000691 aldehyde dehydrogenase 3 family, member A1	12.02	3.89	0.32	0.006

### Ingenuity Analysis

To annotate the sets of genes identified as differentially expressed we used a web-based software, developed by Ingenuity Pathways Analysis [IPA], which derives pathways that are significantly altered by comparing genes identified by SAM against all genes from the CodeLink™ and Illumina™ arrays. Table 5 shows the molecules involved in various networks and their primary functions related to the networks. In Network 1, 14 focus genes were associated with 12 additional genes by direct interactions, which were primarily linked with cell death, neurological disease and cancer. Twelve focus genes were directly linked with 10 other genes in the Knowledge Database, together composing Network 2, primarily associated with cell death, gene expression and cancer. The 7 focus genes linked with 4 other genes involved in Network 3 are associated with dermatological diseases and conditions, cellular growth and proliferation. Networks 4 and 5 consisted of 1 focus gene each but were involved in a wide variety of functions including cardiovascular system development and function, cellular compromise, cellular growth and proliferation, lipid metabolism, nucleic acid metabolism and small molecule biochemistry. Table 6 groups the focus genes based on the cellular function/pathways or disease with which they are involved. This analysis of networks 1, 2, 3, 4 and 5 also revealed tumor necrosis factor (TNF), interferon gamma (IFN γ), mitogen-activated protein kinase 3 (MAPK3), DNA damage inducible transcript 4-like (DDIT4L) and pyruvate dehydrogenase kinase 3 (PDK3) to be the most outstanding partners found within the network.

**Table 5:** Ingenuity analysis comprised of five networks on the genes differentially identified using SAM analysis.

**Networks**	**Associated Network Functions**	**Score**	**Focus Genes**	**Top Functions**
1	AKR1C1, ALDH2, ALDH3A2, CD79B, CLU, FBLN1, GOS2, HSD11B1, IPO7, LAMA4, NPTX2, SERPINB8, SOD2, WTAP	26	14	Cell Death, Neurological Disease and Cancer
2	ADH1B, AIM1, CDO1, CHI3L2, FBXO2, JUP, KHDRBS3, PLA2G1B, PTPRN2, SEPP1, SMAD1, SMCY	12	12	Cell Death, Gene Expression, Cancer
3	COL14A1, MRLC2, MAGPA, NTF3, RGS16, RHOF, SGK	7	7	Dermatological Diseases and Conditions, Cellular Growth and Proliferation
4	DDIT4L	1	1	Cardiovascular System Development and Function, Cellular Compromise, Cellular Growth and Proliferation
5	PDK3	1	1	Lipid Metabolism, Nucleic Acid Metabolism, Small Molecule Biochemistry

**Table 6:** Top canonical pathways identified by ingenuity knowledge based analysis in Dupuytren's Contracture.

**Function**	**Significance (*p *value)**	**Genes**
Bile Acid Biosynthesis	4.96E-04	ADH1B, ALDH2, ALDH3A2
Ascorbate and Aldarate Metabolism	1.11E-03	ALDH2, ALDH3A2
Glycolipid Metabolism	4.8E-03	ADH1B, ALDH2, ALDH3A2
Glycolysis/Gluconeogenesis	6.41E-03	ADH1B, ALDH2, ALDH3A2
Histidine Metabolism	9.42E-03	ALDH2, ALDH3A2

### Gene Products Validated Using Real time RT-PCR

**Figure 1:**
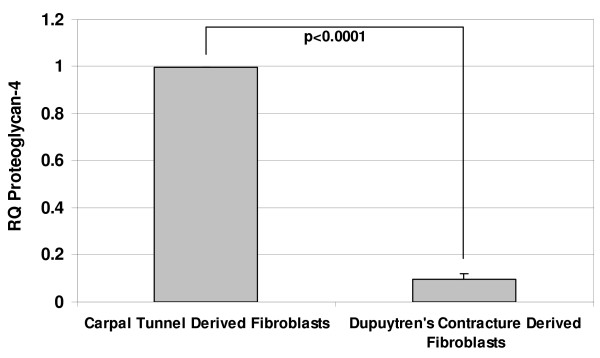
**The relative decrease in gene expression levels for PRG4, FBLN-1 and Type XV collagen in DC derived fibroblasts as determined by real-time RT-PCR.** Values are mean ± SEM of three independent studies, each performed in duplicates. GAPDH was used as an internal control. Statistical analyses were performed by Student's *t *test. Relative quantification of gene expression was calculated by comparing δ C_t _values between carpal tunnel and DC derived fibroblasts.

Real-time RT-PCR was used to confirm the expression levels of three candidate genes: proteoglycan 4 (PRG4), fibulin-1 (FBLN-1) transcript variant D and type XV collagen alpha 1 chain (Figure 1, 2 &3). These genes were all identified as downregulated in the data sets of both microarray based platforms (although SAM analysis picked them out only in one). Real-time RT-PCR results showed that PRG4 mRNA levels in DC fibroblasts was less than 10% of that in control fibroblasts. Similarly the FBLN-1 and collagen genes showed expression levels of less than 40% of those observed in the controls. Thus, in all three cases, the quantitative RT-PCR results confirmed the microarray findings.

**Figure 2:**
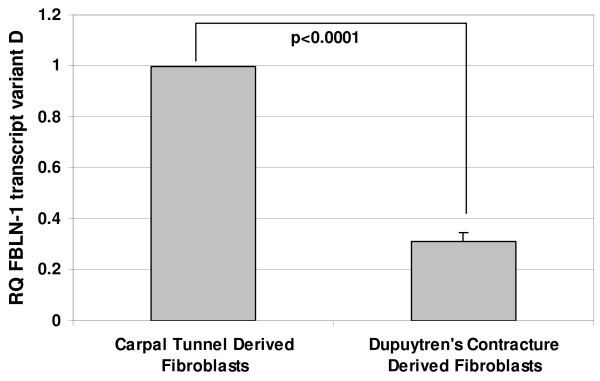
**The relative decrease in gene expression levels for PRG4, FBLN-1 and Type XV collagen in DC derived fibroblasts as determined by real-time RT-PCR.** Values are mean ± SEM of three independent studies, each performed in duplicates. GAPDH was used as an internal control. Statistical analyses were performed by Student's *t *test. Relative quantification of gene expression was calculated by comparing δ C_t _values between carpal tunnel and DC derived fibroblasts.

**Figure 3:**
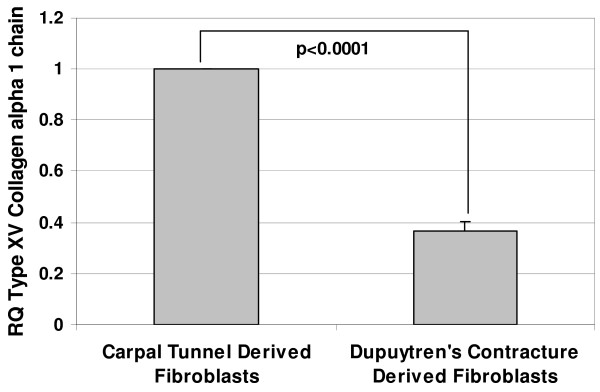
**The relative decrease in gene expression levels for PRG4, FBLN-1 and Type XV collagen in DC derived fibroblasts as determined by real-time RT-PCR.** Values are mean ± SEM of three independent studies, each performed in duplicates. GAPDH was used as an internal control. Statistical analyses were performed by Student's *t *test. Relative quantification of gene expression was calculated by comparing δ C_t _values between carpal tunnel and DC derived fibroblasts.

## Discussion

The microarray analyses of DC fibroblasts versus controls reported here identified far more genes as downregulated in the disease samples than upregulated. This is consistent with previous microarray findings from DC tissues wherein the authors similarly report substantially more downregulated genes than upregulated genes [31], although a countervailing report identified more genes as upregulated than downregulated on tissues derived from DC [24]. The present study using fibroblast cultures did not identify some of the previously reported genes that might be involved in DC, eg. alpha-1 integrins, MafB [25] or various MMPs/TIMPS [26]. This may be due to differences between tissues (where multiple cell types may be present, possibly interacting with each other) and cultured cells expressing a solitary phenotype. Our results across two microarray platforms are internally consistent and indicate that fibroblast cell populations in DC generally preferentially downregulate selected gene expression relative to control cells.

Dupuytren's disease presents two fibrotic structures that are clearly identifiable: the nodule and the cord [32,4]. The nodule is a relatively vascular tissue containing a dense population of fibroblasts, with a high proportion being myofibroblasts. In contrast, the cord is a collagen-rich structure that is relatively avascular and acellular, and with a lesser (but still significant) abundance of myofibroblasts. The slow progression of the disease, combined with a lack of awareness as to eventual severity and that surgical resection is rarely performed in the absence of pronounced finger contracture, limits the availability of nodule tissue for experimental study.

In the present study, we identified by microarray analyses candidate genes from cord-derived fibroblasts that might serve as targets for intervention to limit the recurrence of palmar fascia fibrosis. One of the hallmarks of DC is abnormal proliferation of fibroblasts [33,34] with excessive production of collagen (predominantly type III collagen) along with elevated levels of other ECM proteins, mainly fibronectin, tenascin and laminin [17,35,36]. Higher expression of laminin and tenascin has been reported in nodular tissues [35] and an elevated level of fibronectin expression has been found in both nodular tissues and in cord derived fibroblasts [36]. Since alterations in ECM proteins appear to play an important role in DC, we looked for differences in the gene expression patterns of several ECM proteins between normal and diseased derived palmar fascia fibroblasts. Intriguingly, we did identify changes in the expression of other ECM proteins including, types XIV and XV collagens, PRG4 and FBLN1 transcript variant D which have not been previously associated with DC. The three fibroblast-derived ECM genes were initially identified through SAM analysis of the Illumina™ data, but were not identified by SAM using the CodeLink™ data. However, direct inspection of the CodeLink raw data did show a statistically significant downregulation of these genes that was confirmed by real-time RT-PCR analysis (Table 4 & Figure 1, 2 &3).

Milani et al., (1994) [37] reported that type XV collagen (undulin) is constitutively expressed in normal hepatic liver, but that its levels are elevated in fibrotic liver, suggesting its participation in the rearrangement of connective tissue during fibrotic disease. Since DC is a fibroproliferative disease, high levels of undulin might be expected. Our analysis however, indicates that type XV collagen mRNA levels are relatively lower in Dupuytren's-derived fibroblasts, suggesting that overexpression of this gene product is not universal in fibrotic disorders but may vary with tissue type and location. The downregulation of type XV collagen in DC-derived fibroblasts warrants further study as it has been shown previously that lack of type XV collagen results in skeletal myopathy and cardiovascular defects in mice, demonstrating that type XV collagen can prove a significant contributor in the pathogenesis of musculoskeletal disorders [38].

We also identified two other ECM proteins, PRG4 and FBLN1, as being downregulated in DC-derived fibroblasts. PRG4, also commonly referred to as megakaryocyte stimulating factor, articular superficial zone protein and lubricin, is a multifaceted cytoprotective glycoprotein that independently or additively contributes to boundary lubrication in synovial joints and at articular cartilage-cartilage interfaces [39,40]. Recently Alazami et al., (2006) [41] have corroborated the earlier findings of Marcelino et al., (1999) [42] demonstrating that camptodactyly-arthropathy-coxavara-pericarditis (CACP) syndrome (where one or more fingers are curved inwards on the palm (flexed) and cannot be straightened) is an autosomal recessive disorder caused by mutations in the PRG4 gene. Moreover, PRG4 deficiency has also been implicated in the pathogenesis of osteoarthritis [43], thus making this an attractive candidate gene for further study. The fibulin-1 gene, which encodes both an extracellular matrix protein and a secreted plasma glycoprotein, has been found to be disrupted in a chromosomal translocation (12;22) which results in haploinsufficiency for the FBLN1-D variant in synpolydactyly, a congenital condition characterized by a union of fingers or toes and/or increase in the number of toes or fingers. Biochemical analyses of synpolydactyly fibroblasts showed significantly reduced levels of FBLN1-D polypeptide incorporated in the ECM [44]. In summary, PRG4, fibulin-1 and type XV collagen are all potential candidates for being involved in the pathogenesis of DC and the biological role of these ECM proteins in this disease warrants further study. Interestingly, Ingenuity Knowledge Based Analysis identified fibulin-1 as a focus gene; it is one of the many genes included in Network 1 and appears to have interaction with other ECM proteins, namely laminin 4, versican (chondritin sulfate proteoglycan 2) along with the transcription factor Sp1 (SP1) and connective tissue growth factor (CTGF).

In addition to the genes discussed above, we identified several others that produced concordant results on the two microarray platforms. These included three metabolic genes involved in alcohol metabolism: aldehyde dehydrogenase 2 family (ALDH2), aldo-keto reductase family, member C1 (AKR1C1), aldehyde dehydrogenase 3 family, member A1 (ALDH3A1), all of which were downregulated in the DC palmar fascia fibroblasts relative to controls and observed previously by Pan et al. (2003) [31]. ALDH2 was identified as a focus gene and listed as a target in various signaling cascades (Table 6) through Ingenuity Knowledge Based Analysis. The analysis also suggests that TNF might increase the expression of ALDH2. The data provides further evidence that these metabolic genes have the potential to play a role in the progression of DC. In our studies, ALDH2 and AKR1C1 were identified by both platforms, whereas ALDH3A1 was not identified by the CodeLink™ platform. However, a close examination of the data points for ALDH3A1 in the CodeLink™ data set does reveal a statistically significant reduction (p < 0.005) with greater than a 3-fold change between the two study groups (Table 4), albeit not enough to satisfy the extremely stringent parameters of SAM analysis. The downregulation of these three genes involved in alcohol metabolism is congruent with the observation that alcohol consumption is linked with Dupuytren's disease.

There is also some evidence to show that a relationship exists between the pathogenesis of Dupuytren's contracture induced by various oxidative stress molecules, including superoxide radical (O_2-_), hydrogen peroxide (H_2_O_2_) and the hydroxyl radical (OH-) [45,46]. It is hypothesized that the progressive restriction of capillaries with age, smoking and other environmental factors leads to a condition of localized hypoxia resulting in increased levels of xanthine oxidase and the subsequent production of free radicals. In the present study, we found mitochondrial superoxide dismutase 2, cysteine dioxygenase type 1, and selenoprotein P, plasma, 1 (SEPP1) to be downregulated in DC-derived fibroblasts. Low levels of these enzymes may render DC-derived fibroblasts deficient in their ability to detoxify superoxide radicals or to oxidize cysteine residues as an antioxidant defense mechanism [45,47]. Clusterin, a secreted mammalian chaperone associated with stress-associated cell survival (anti-apoptotic gene) [48] is also downregulated in DC, contrasting with its overexpression in several human cancers such as prostate, breast, and squamous cell carcinoma [49,50].

Chitinase-3-like 2 (CHI3L2), a secreted glycoprotein component of the ECM which has been linked to early detection and prognosis of ovarian cancer, was identified in this study. CHI3L2 appears to be downregulated in DC as opposed to increased expression seen in ovarian cancer patients [51]. Angiopoietin-like factor (CDT6), a homologue to the angiopoietin gene family, is a glycoprotein that is involved in vascular morphogenesis and maintenance through binding to the vascular endothelial Tie2 receptor [52]. CDT6 is also found to be downregulated in DC-derived fibroblasts. The significance of altered CHI3L2 and CDT6 expression to the progression of Dupuytren's contracture is as yet unclear.

The finding that serum/glucocorticoid regulated kinase (SGK) message is downregulated in DC fibroblasts is contrary to previous studies that document a high level of expression of SGK transcript in fibrosing disorders such as Crohn's disease, fibrosing pancreatitis, diabetic nephropathy, lung fibrosis and liver scirrhosis [53]. F-box protein 2 (FBXO2), which plays a crucial factor in the ubiquitin-mediated degradation of cellular regulatory proteins [54], is also noted to be downregulated in DC-derived fibroblasts. Hydroxysteroid (11-beta) dehydrogenase transcript variant 1 catalyzes the interconversion of biologically inactive glucocorticoid (cortisone) to active glucocorticoid (cortisol); it has been shown to play a predominant role in obesity, type 2 diabetes [55] and hypertension but its role in fibrotic disease is yet to be determined.

Of interest is the observation that 29 of the dysregulated genes identified in the DC fibroblasts are of unknown function, suggesting that entirely new pathways may be operational here that have not been previously characterized. An understanding of these genes and their protein products may provide new insights into DC and other disorders potentially associated with DC. In summary, our study reveals a variety of candidate genes, some of known function, some of unknown function, that together with additional genetic studies will point the way to a better understanding of this most common of human connective tissue disorders.

## Conclusion

The present study using fibroblasts derived from DC patients has identified a variety of candidate genes that may be involved in the pathophysiology of palmar fibrosis which may ultimately serve as attractive molecular targets for alternative therapies. Future studies will further correlate the changes in the mRNA levels to the protein expression both at the cellular and tissue level which will provide further clues to intervene and limit the progression/recurrence of DC.

## Competing interests

The authors declare that they have no competing interests.

## Authors' contributions

LS contributed to the conception and design of the study, acquisition and interpretation of the data and drafting the manuscript. SK contributed to the conception and design of the study, interpretation of the data and critically revised the manuscript for important intellectual content. SJ, BJ and FZH contributed to the acquisition of the data. DO'G and WAL participated in the analysis and interpretation of the data and critically revised the manuscript. BSG collected the samples used and critically revised the manuscript. MAB contributed to the design of the study and critically revised the manuscript. JCP and GDE contributed to the design of the study, provided financial support, and critically revised the manuscript. All authors read and gave final approval of the manuscript.

## Pre-publication history

The pre-publication history for this paper can be accessed here:


